# Challenges in identifying the best source of stem cells for cardiac regeneration therapy

**DOI:** 10.1186/s13287-015-0010-8

**Published:** 2015-03-13

**Authors:** Parul Dixit, Rajesh Katare

**Affiliations:** Department of Physiology, HeartOtago, Otago School of Medical Sciences, University of Otago, Dunedin, 9010 New Zealand

## Abstract

The overall clinical cardiac regeneration experience suggests that stem cell therapy can be safely performed, but it also underlines the need for reproducible results for their effective use in a real-world scenario. One of the significant challenges is the identification and selection of the best suited stem cell type for regeneration therapy. Bone marrow mononuclear cells, bone marrow-derived mesenchymal stem cells, resident or endogenous cardiac stem cells, endothelial progenitor cells and induced pluripotent stem cells are some of the stem cell types which have been extensively tested for their ability to regenerate the lost myocardium. While most of these cell types are being evaluated in clinical trials for their safety and efficacy, results show significant heterogeneity in terms of efficacy. The enthusiasm surrounding regenerative medicine in the heart has been dampened by the reports of poor survival, proliferation, engraftment, and differentiation of the transplanted cells. Therefore, the primary challenge is to create clearcut evidence on what actually drives the improvement of cardiac function after the administration of stem cells. In this review, we provide an overview of different types of stem cells currently being considered for cardiac regeneration and discuss why associated factors such as practicality and difficulty in cell collection should also be considered when selecting the stem cells for transplantation. Next, we discuss how the experimental variables (type of disease, marker-based selection and use of different isolation techniques) can influence the study outcome. Finally, we provide an outline of the molecular and genetic approaches to increase the functional ability of stem cells before and after transplantation.

## Introduction

An estimated 17 million people each year die of cardiovascular diseases, particularly heart attacks and strokes. In addition, cardiovascular diseases are also a cause of lifelong disabilities and a reduction in the productive years of life. The most common form of heart disease is ischaemic heart disease (IHD), where there is an imbalance between myocardial oxygen supply and its demand. This often leads to disturbances in impulse formation and conduction in the heart in the form of arrhythmias and, if the ischaemia is sustained, necrosis of the heart muscle (myocardial infarction (MI)) may develop [1].

**Figure 1:**
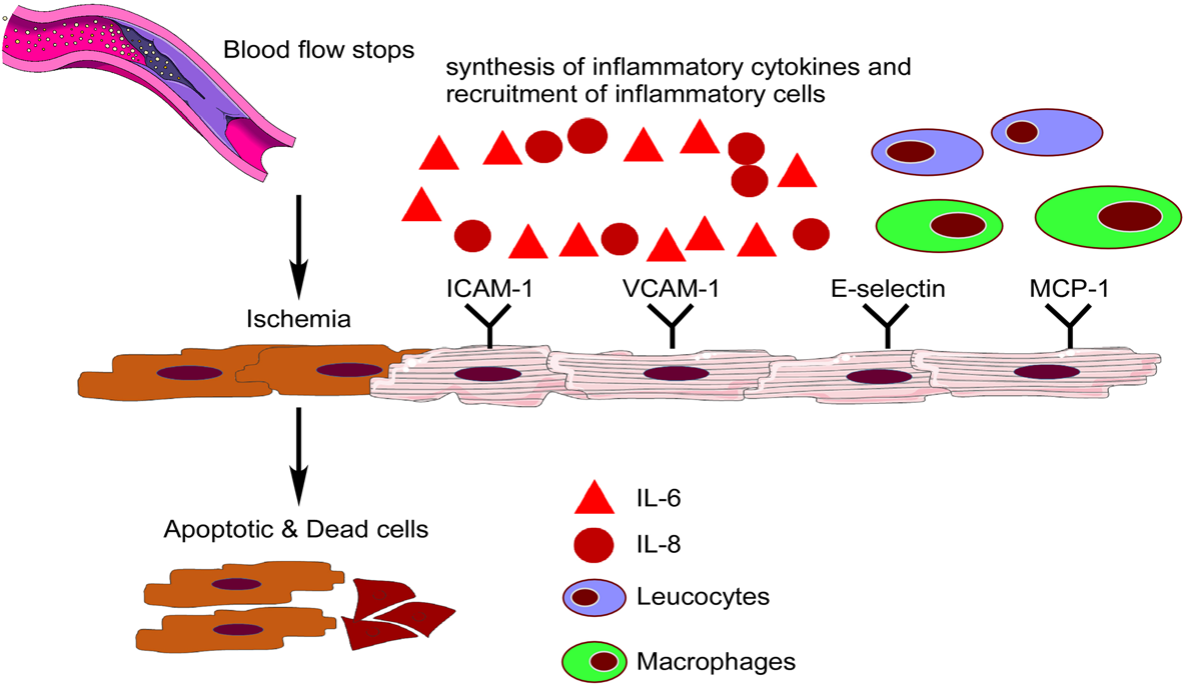
**Inflammatory response in the heart during ischaemia.** ICAM-1, intercellular adhesion molecule-1; IL, interleukin; MCP-1, monocyte chemo-attractant protein-1; VCAM-1, vascular cell adhesion molecule-1.

The innate response of the heart to an ischaemic insult has a deleterious as well as a protective effect. An acute response involves the synthesis of inflammatory mediators, cytokines such as tumour necrosis factor-α, monocyte chemo-attractant protein-1, and interleukin (IL)-1β, IL-6, and IL-8 and the up-regulation of cell adhesion molecules such as E-selectin, intercellular adhesion molecule-1, and vascular cell adhesion molecule-1. This is followed by an invasion of monocytes, leukocytes, and macrophages at the site of injury (Figure 1) [2,3]. There is also an accumulation of dead tissue, metabolites, and cellular debris. Ultimately, a necrotic zone is formed in the heart, which, in due course, leads to functional abnormalities, such as reduced myocardial contractility and diastolic dysfunction. Eventually, the surviving myocardium hypertrophies and myofibroblasts infiltrate the injury site.

The adaptive response of the heart to this ischaemic insult is the activation of pathways that increase oxygen delivery and promote pro-survival responses. This is made possible by the increased expression of proteins such as erythropoietin, vascular endothelial growth factor, insulin-like growth factor 2, and glucose transporter [2]. Neovascularisation occurs in an effort to resupply the ischaemic zones with blood and is initiated by the release of soluble stromal cell-derived factor-1 (SDF-1), which is a ligand for C-X-C chemokine receptor type 4 (CXCR4), a receptor on many endothelial progenitor cells (EPCs) [4].

Based on this evidence, the long-term holistic treatment of IHD necessitates a therapy which mimics and magnifies the heart’s endogenous protective response. Currently, the standard treatment for people with IHD is surgical intervention with primary angioplasty and/or the introduction of a stent or a coronary artery bypass graft (CABG). The use of primary angioplasty and stents to reopen the blocked artery has resulted in a 33% reduction in the mortality rate in patients with IHD. Besides surgical procedures, pharmacological treatments such as coronary vasodilators, anti-coagulants, and anti-platelet agents also delay the onset of heart failure [5]. However, pharmacological and surgical therapies cannot make up for the loss of myocytes. The only standard therapy for heart failure that addresses the fundamental problem of cardiomyocyte loss is cardiac transplantation, but organ transplantation is not always a feasible option as the number of patients with end-stage cardiac failure is far greater than actual availability of suitable donors [6].

**Figure 2:**
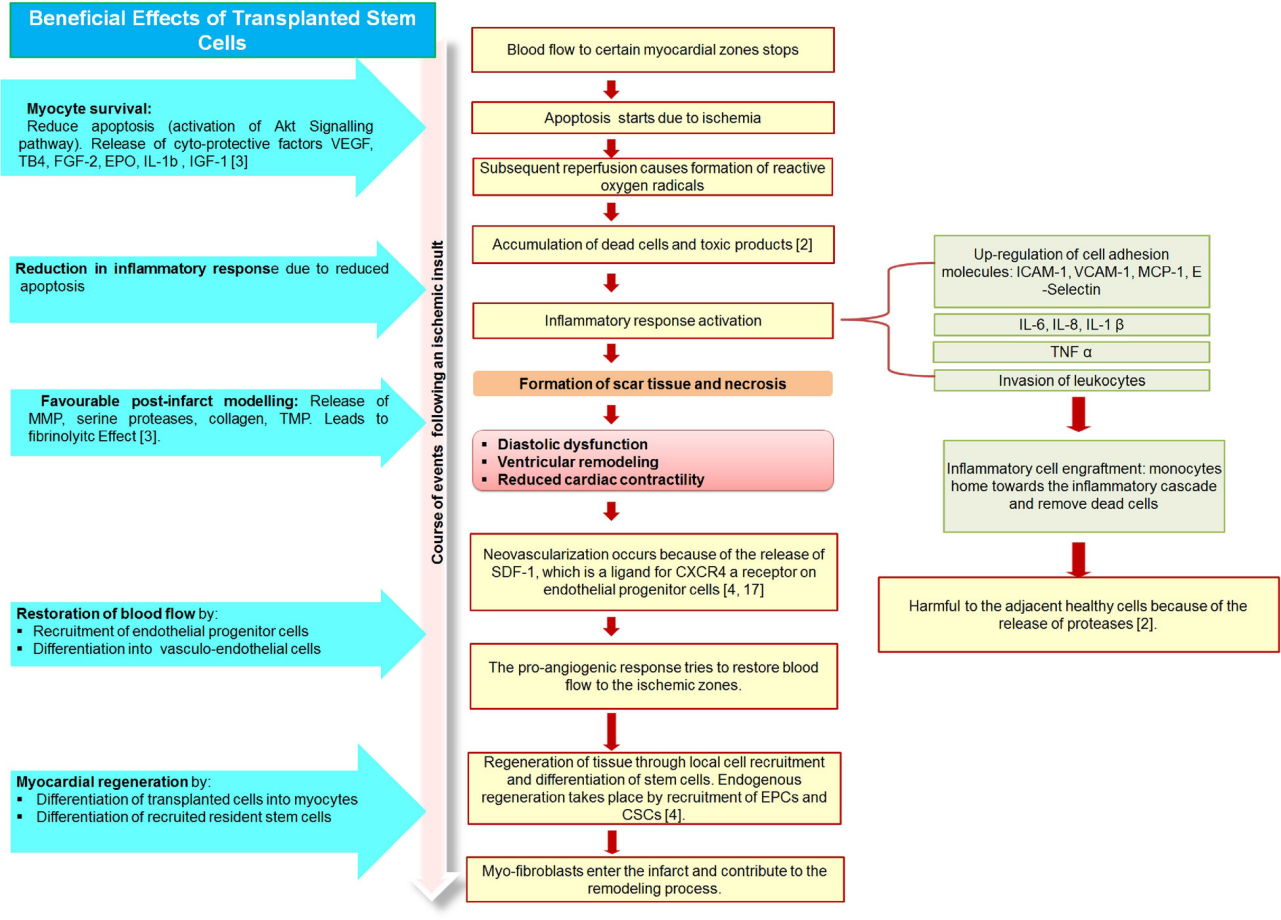
**Beneficial effect of stem cells in ischaemic heart disease.** CSC, cardiac stem cell; CXCR4, C-X-C chemokine receptor type 4; EPC, endothelial progenitor cell; EPO, erythropoietin; FGF-2, fibroblast growth factor 2; ICAM-1, intercellular adhesion molecule-1; IGF-1, insulin-like growth factor 1; IL, interleukin; MCP-1, monocyte chemo-attractant protein-1; MMP, matrix metalloproteinase; SDF-1, stromal cell-derived factor-1; TB4, thymosin beta-4; TMP, transmembrane protease; TNF, tumour necrosis factor; VCAM-1, vascular cell adhesion molecule-1; VEGF, .

The ongoing experiments and clinical trials conducted to test the regenerative potential of stem cells in the past decades suggest that stem cell therapy can fulfil most of these demands. Moreover, it provides an all-inclusive approach for the treatment of IHD and heart failure (Figure 2) [7]. Preliminary efficacy studies indicate that stem cells have the potential to enhance myocardial perfusion and/or contractile performance in patients with IHD, (a) by transdifferentiation into cardiomyocytes or vascular cells and (b) through paracrine effects by secreting growth factors which stimulate the repair and growth of host cells and the recruitment of endogenous stem cells [8].

## Stem cells from different sources in the treatment of cardiovascular disease

For regenerative therapy, various cell types at different developmental stages, including embryonic, foetal, and adult cells, have been considered for transplantation into the heart. Each cell type will be discussed in detail in the following sections.

### Human embryonic stem cells

Embryonic stem cells (ESCs) have the capacity to divide indefinitely and differentiate into any cell type. Although ESCs are incapable of spontaneous differentiation into cardiomyocytes, they can be directed to differentiate into cardiomyocytes or cardiac progenitor cells using various induction methods [9]. One established advantage of the use of ESCs is the ability of ESC-derived cardiomyocytes (ESC-CMs) to electrically integrate with the heart muscle. In a swine model of an atrioventricular block, transplanted human ESC-CMs showed electrical coupling and a reversal of the block [10].

One of the initial technical challenges faced in ESC research was the attainment of high purity and a large yield of differentiated cells belonging to a single lineage type [11]. Various approaches, such as genetic modification, specialised culture methods, and treatment with chemical and biological factors, have been used to enrich, purify, and select homogeneous and functionally intact populations of ESC-CMs generated from heterogeneous ESCs [10,12,13]. Recently Chong and colleagues [14] succeeded in generating cardiomyocytes from ESCs on a large scale. These ESC-CMs were able to successfully engraft and repair the injured myocardium in a primate model of myocardial infarction. These results are encouraging, since only very few cell types have shown efficacy in large animals. Importantly, ESCs are pluripotent cells, which give them an advantage over other adult stem cell types with limited differentiation potential.

Despite the evidence of ESCs’ efficacy in larger animal models, their clinical use has been hampered by important limitations, including their genetic instability, potential tumorigenic and immunogenic properties, and ethical considerations related to the origin of these cells [15]. In addition, few European nations, for example, have strict laws prohibiting ‘destructive embryo research’, while federal laws in the USA permit the use of embryos which have been discarded after *in vitro* fertilisation [16].

### Skeletal myoblasts

Skeletal myoblasts, also referred to as skeletal muscle satellite cells, were one of the initial few cell types first considered for cardiac regeneration [17]. These cells are a type of progenitor cell with myogenic capacity, and they are abundantly expressed in the human body. Isolated skeletal myoblasts can be made to proliferate and expand *in vitro*. The other advantages of these cells are their ability to contract and their capacity to withstand ischaemic insult [18,19].

The evidence of efficacy in animal models facilitated the use of skeletal myoblasts in clinical trials. Myoblast Autologous Grafting in Ischemic Cardiomyopathy (MAGIC) was the first randomised placebo-controlled study of myoblast transplantation. However, this study did not demonstrate an incremental improvement in left ventricular function over that provided by CABG alone [20]. Later, multiple experiments were aimed towards trans-differentiating these cells into cardiomyocytes, but none of the attempts were successful, suggesting that skeletal myoblasts are committed towards a skeletal muscle fate [18].

Moreover, myofibres derived from the transplanted skeletal myoblasts fail to integrate electromechanically with the host myocardium due to a lack of adhesion proteins. This results in the failure to develop the intercalated discs required for electrical integration between adjacent myofibres. The excitement around skeletal myoblasts was further reduced when clinical studies reported the occurrence of ventricular arrhythmias following transplantation in patients. Failure to electrically couple these cells with the existing tissue may be a reason for myofibres’ arrhythmogenicity. Genetic modification to introduce the expression of connexin 43, a gap junction protein, was considered as a strategy for overcoming this limitation [21]. However, a later study by Fernandes and colleagues [22] found that this modification was not sufficient to reduce the arrhythmogenic potential of these cells.

### Bone marrow stem cells

The first research on stem cells was conducted on bone marrow as early as 1950, which found at least two kinds of stem cells within bone marrow: hematopoietic stem cells (HSCs) and mesenchymal stem cells (MSCs). The ability of transplanted bone marrow-derived cells (BMCs) to regenerate the infarcted myocardium was first shown by Orlic and colleagues in 2001 [23]. They demonstrated that HSCs marked by the surface protein c-kit were accountable for the trans-differentiation of BMCs into mature cardiomyocytes, smooth muscle cells, and endothelial cells in a murine model of MI. In support of the above results, injection of isolated c-kit^+^ cells into the peri-infarct regions resulted in improved left ventricular function in the infarcted heart [23]. However, their claim regarding the ability of HSCs to transdifferentiate into cardiovascular cells has been questioned by several other studies [24-27]. Nevertheless, the important outcome was that these cells showed a significant improvement in cardiac function after engraftment. Apart from c-kit, many other cell surface markers have also been identified that define populations enriched for freshly isolated human HSCs, including the CD133^+^ and CD34^+^ hematopoietic cells [28]. Interestingly, c-kit^+^ HSCs have never been tested clinically, which is required to truly compare their efficacy with other cell types. Similar to the c-kit^+^ cells, CD34^+^ cells have also been considered for cardiac regeneration. CD34^+^ cells are routinely used clinically to reconstitute the deficient hematopoietic system after radiation or chemotherapy [29]. In addition to being a resident population in the bone marrow, these cells were identified in the peripheral blood by Körbling and colleagues [30]. Evidence has suggested that EPCs and differentiated endothelial cells also express CD34, leading to studies testing the angiogenic capacity of bone marrow and peripheral blood-derived CD34^+^ cells. These cells also showed the ability to differentiate into cardiomyocytes and smooth muscle, in addition to endothelial cells, after transplantation into an infarcted heart [31]. However, this approach was questioned by Norol and colleagues [32], who did not find any cardiac phenotype following transplantation of CD34^+^ cells into non-human primates.

Despite these controversial findings, most clinical trials to date have used total bone marrow mononuclear cells, which comprise HSCs, MSCs, and monocytes [33]. A review by the Cochrane Heart Group summarised 33 clinical trials (1,765 patients) on the effectiveness of BMCs for cardiac regeneration following acute MI and concluded that while no significant improvement was observed in the mortality and morbidity of the patients who received BMCs, they demonstrated a significant and sustained improvement (12 to 61 months follow-up period) in left ventricular ejection fraction (LVEF) [34]. Further, in a recent meta-analysis (23 clinical trials and 1,255 patients) the same group concluded that, in addition to the improvement in LVEF, BMCs were also able to improve the morbidity and mortality in patients with chronic IHD and congestive heart failure [35]. Despite these promising results, debate continues about whether the therapeutic potential of BMCs in improving left ventricular function might be attributed mostly to its paracrine effects [36].

The conclusions from the Cochrane Heart Group were optimistic but caveats included the high degree of heterogeneity observed in the results. A recent study utilised weighted regression and meta-analysis to compare the results from 49 trials using BMCs for cardiac regeneration [37]. This analysis identified 600 discrepancies in 133 reports from these trials. Interestingly, the trials with the highest number of discrepancies also showed the maximum increment in LVEF in patients. The studies that failed to show any benefit from BMCs had the lowest number of discrepancies [37]. With such differing data available from clinical trial results, it is extremely difficult to draw conclusions on the efficacy of BMCs [38].

### Mesenchymal stem cells

MSCs have been typically considered as the cells with the capacity for self-renewal, and differentiate into the mesenchymal lineages, including skeletal myoblasts, chondrocytes, and adipose tissue [39]. This classical view is now challenged as they have been shown to differentiate into neural (non-mesenchymal) tissues as well [40] [39]. The adherent MSC population is shown to express cell surface markers CD73, CD105, CD29, CD44, and CD90 and lack CD34 and CD45, which are mainly expressed by HSCs [41]. Considering that these cells can be isolated from a variety of tissues, including bone marrow, adipose tissue, and cord blood, it makes them a more practical option for regenerative therapy. The second advantage with MSCs is that they lack major histocompatibility complex II and B7 co-stimulatory molecule expression; hence, they are able to evade immune responses and have an innate ability to overcome the rejection. This opens the possibility of non-autologous transplantation in patients [39,42].

MSCs have been shown to differentiate into cardiomyocytes as well as vascular endothelial cells *in vitro* [39]. Conversely, experimental evidence suggests that when transplanted *in vivo*, MSCs contribute to neo-vascularisation and cardiomyocyte protection, mainly through the activation of paracrine factors. They may persist within the myocardium in a differentiated state, although substantial evidence for their ability to attain cardiac cell phenotype *in vivo* is still needed [43].

### Induced pluripotent stem cells

The cellular differentiation process was once believed to be an irreversible process. In 2006, however, Takahashi and Yamanaka [44] successfully induced pluripotency in somatic cells through retroviral transduction of several factors involved in the self-renewal of ESCs. The combination of transcriptional factors commonly used for cellular reprogramming are Krüppel-like factor 4 (Klf-4), sex determining region Y-box 2 (Sox-2), c-Myc or octamer-binding transcription factor 4 (Oct3/4), Nanog, and Lin-28 [44]. Since then, several studies have demonstrated the wide differentiation potential of induced pluripotent stem cells (iPSCs), which includes their ability to differentiate into cell types from any of the three germ layers [45]. iPSCs have been found to differentiate into cardiomyocytes, endothelial cells, and smooth muscle cells *in vitro*. When injected into the infarcted heart of mice, iPSCs can differentiate into the cardiac phenotype [46,47].

One of the initial problems with using iPSCs was the poor experimental efficiency in the successful induction of pluripotency to somatic cells. The use of genetic factors, chemical inhibitors, and signalling molecules that can either replace core reprogramming factors or enhance reprogramming efficiency has now been investigated. Recently, Rais and colleagues [48] found that the Mbd3/NuRD (nucleosome remodelling and de-acetylation) repressor complex is the predominant molecular block preventing the deterministic induction of ground-state pluripotency, and hence by depleting the *Mbd3* gene, they could successfully synchronise all cells to attain pluripotency. This does overcome a chief barrier to the clinical use of iPSCs [48]. Due to their ESC-like properties, however, they were also found to be tumorigenic [49]. Hence, to overcome the problem of tumorigenesis, Martens and colleagues [46] differentiated iPSCs into cardiomyocytes *in vitro* before transplanting them into the infarcted heart. Transplanted cells not only improved the cardiac functions, but also localised to the host myocardium [46]. Further, biosynthetic tissues are also created from cardiac cells derived from iPSCs [50]. Several bioengineering strategies are being explored to improve the efficacy of iPSC-derived transplants to improve their engraftment, survival, and functionality in tissues [51]. The *in vivo* safety and functionality of these cells need to be assured before their clinical translation is considered.

### Endogenous cardiac stem cells

Until a decade ago, most research in cardiology was influenced by the dogma that the heart is a terminally differentiated organ and is incapable of generating new parenchymal cells. Hence, the only response of cardiomyocytes to stress was considered to be either hypertrophy or death. However, evidence has shown that myocytes undergo replication, mitotic division, and spontaneous regeneration in the heart [52]. To further discredit the view that the heart is a post-mitotic organ, research has established the presence of a pool of resident cardiac progenitor cells and cardiac stem cells (CSCs) expressing the stem cell surface marker c-kit in the adult rat (and human) heart [53]. The new dynamic view considers that cell death and cell restoration in the heart are a part of organ homeostasis, although the rate of myocyte renewal/turnover is very low [54]. A cardiac progenitor cell is an immature but already committed cardiac cell that can proliferate and mature into precursors which, in turn, develop into one of the main matured cardiac cell types. CSCs are a heterogenic group of cells and are concentrated in specific areas of the heart, such as the atria or pericardium [54]. Other populations of stem cells found in the heart are side population cells (which are identified based on their ability to exclude Hoechst dye), stem cell antigen-1, and islet-1 transcription factor expressing cells [55,56]. In addition, CSCs have also been demonstrated to express MSC markers such as CD90 and CD105 and ESC markers Rex1, Nanog and Sox2 [57]. Apart from this, CSCs have also been identified based on the expression of early cardiogenesis markers such as platelet derived growth factor receptor-α, and foetal liver kinase-1 [58].

Cardiac stem cell research is a rapidly emerging research area with many stem cell-like populations being newly discovered in the heart. For example, a recent study indicated a significant contribution of embryonic epicardial progenitor cells to the cardiomyocyte lineage [59]. Due to the lack of clearly defined markers, epicardial derived cells have not been tested rigorously for their therapeutic efficacy, although recent characterisation of these cells based on the specific marker Wilms tumour-1 could lay the foundation to further studies [60].

Similarly, a population of adult epicardial-resident cardiac colony-forming unit fibroblasts isolated by Chong and colleagues [61] displayed broad trans-germ layer potency *in vitro* and *in vivo*. This is a promising cell type because, unlike the discovery of c-kit-positive cells, rigorous gene expression and fate lineage analysis was used to characterise this population. Epicardial-derived cells might hold the true potential for cardiac regeneration owing to their role in embryonic cardiogenesis and multiple cardiac lineage differentiation capacities. Still, a deeper dissection of the role of these cells in homeostasis and repair is warranted before they are tested clinically. Unlike BMCs, for which surface markers have been extensively characterised, the resident CSCs and cardiac progenitor cells show a mixed and overlapping expression of stem cell markers [62].

One of the distinctive features making CSCs a good candidate in cardiac regeneration is their cardiac commitment and ability to undergo consistent cardiomyogenic and angiogenic differentiation. CSCs from small sized human myocardial biopsies can be clonally expanded up to many fold *in vitro* [53,63]. CSCs may be preferable over cells from other lineages (like BMCs) as they have been shown to reach functional competence and obtain the structural characteristics of mature myocytes and vessels faster than BMCs [64].

Many animal studies have documented the ability of clonally expanded CSCs to improve heart function following transplantation in animal models of MI [65]. The bulk of pre-clinical studies conducted present substantial proof of clonally expanded CSCs’ regenerative ability and have paved the way for clinical trials. Stem Cell Infusion in Patients with Ischemic Cardiomyopathy (SCIPIO) is an ongoing first-in-human, randomised, open-label trial of autologous c-kit^+^ CSCs in patients with heart failure due to IHD undergoing CABG. The initial data obtained from this trial looked encouraging and compared favourably with prior studies on intracoronary bone marrow mononuclear cell infusion in a similar patient population [66,67]. However, the *Lancet* (which published the SCIPIO trial results) has expressed concern over the integrity of certain data published in the SCIPIO trial, although the issue remains under review and should not be pre-judged prior to completion of the investigation [68].

In the CArdiosphere-Derived aUtologous stem CElls to reverse ventricUlar dySfunction (CADUCEUS) study, 31 patients with acute MI who had undergone successful coronary angioplasty but were left with reduced cardiac function were randomised to receive standard care or autologous cardiosphere-derived cells (CDCs) [69]. CDCs are a natural mixture of stromal, mesenchymal, and progenitor cells and are derived from the culture of percutaneous endomyocardial biopsies, which yield spherical multicellular clusters called ‘cardiospheres’. From these cardiospheres, millions of proliferative cells that express markers of stromal, mesenchymal, and progenitor cell-related antigens, as well as other cells undergoing spontaneous cardiac differentiation, could be harvested [70]. The patients treated with CDCs showed a reduction in scar mass, increased viable heart mass and improved regional contractility [69].

The clinical trials accomplished two crucial goals. Firstly, they indicated the possibility of culturing therapeutic doses of autologous CSCs from a small amount of myocardial biopsy tissue. Secondly, these cells could be successfully administered by intracoronary injection to patients with prior MI [67,69,71]. For autologous transplantations, CSCs are usually isolated from atrial appendages and ventricular and epicardial biopsies [72]. However, since atrial and ventricular cardiomyocytes have differential gene expression and functional efficacy, it is not known whether the stem cells from atria and ventricles have the same characteristics and functions [73].

While many of the preclinical and clinical trials, including the above mentioned SCIPIO trial, used c-kit^+^ cells as the primary source of myocardial regeneration after injury, van Berlo and colleagues [74] recently showed that cardiomyocytes generated by c-kit^+^ cells *in vivo* are functionally insignificant. In contrast, they demonstrated an ample increase in the number of cardiac endothelial cells by c-kit^+^ cells. These new findings tempt to speculate that the modest improvements seen in the heart with these cells in the clinical trial are due to the ability of c-kit^+^ stem cells to cause the growth of capillaries, which improves vascularisation, rather than the generation of new cardiomyocytes [74].

While all the available evidence demonstrates the heterogeneity of the CSC population, one important question remaining to be addressed is whether the distinct classes of CSCs have inherently distinct roles in cardiac regeneration.

## Challenges in identifying the best source of stem cells for cardiac regeneration

**Figure 3:**
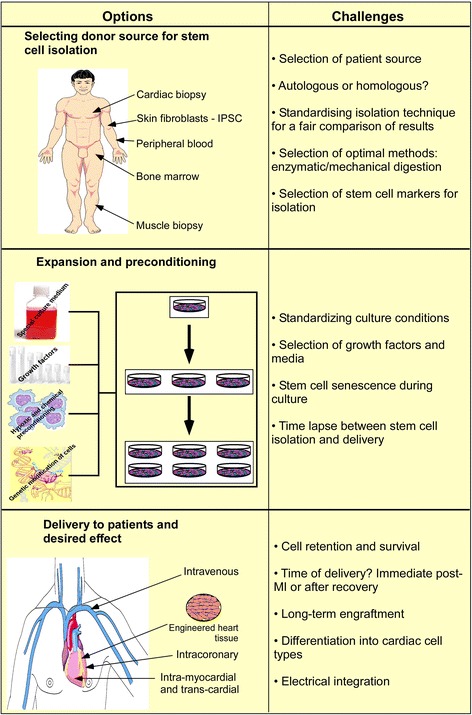
**Challenges in stem cell delivery.** iPSC, induced pluripotent stem cell; MI, myocardial infarction.

The overall clinical cardiac regeneration experience suggests that stem cell therapy can be safely performed. However, it also suggests that stem cells can be effectively used only when there are reproducible results, indicating that it is very important to select the right type of stem cell in the right clinical setting. Hence, before translation of stem cell use from preclinical studies to the clinic, one significant challenge is the identification and selection of the best suited stem/progenitor cell types (Figure 3). The primary task is to obtain clear evidence on what truly drives the improvement in heart function after the administration of stem cells. The mechanism(s) underlying the observed functional improvement in the heart remains unclear and is an issue for debate. Classically, it is believed that an ideal stem cell should differentiate into cardiomyocytes that integrate both mechanically and electrically with innate myocytes and should be able to form blood vessels to boost the blood supply to the scar zone. On the other hand, recent studies suggest that paracrine factors secreted by the stem cells may play a more important role in the improvement of cardiac function [75]. This has changed the belief that the stem cells must differentiate into cardiac cell types to improve cardiac function. Hence, stem cell types which are not multi-potent themselves can still improve cardiac function comparable to stem cells committed to cardiac lineages [76]. The next step now is to systematically characterise these cells by their ability to differentiate into cardiac cell types and their ability to improve cardiac health by paracrine mechanisms [8].

### Practical and technical challenges

#### Stem cell source

Many of the cell types considered are pluripotent. The stem cells that are committed to the myocardial lineage can be selected using different reporter systems linked to the endogenous activation and expression of cardiogenic or myogenic genes. For cardiac regeneration, it is essential that the selected population of cells shows high cardiogenic potential, since the injection of highly proliferative uncommitted pluripotent stem cells can lead to carcinogenicity. Due to the large amount of heterogeneity in the number and quality of adult stem cells in patient tissue samples, selecting and isolating a pure population of cardiac lineage committed stem cells is essential for the safety as well as efficacy of stem cell therapy [77].

The number and efficacy of stem cells have been shown to change depending on several factors such as age, gender, treatments, and pre-existing conditions. Sanada and colleagues [78] reported a marked decline in the c-kit^+^ population with an increase in age. This was supported by another study showing a loss of cardio-protective effects in CSCs isolated from older patients, which also achieved early senescence during *in vitro* culture [79]. In addition to age, CSCs isolated from patients with pre-existing morbidities, such as diabetes and hypertension or end-stage heart disease, also showed marked differences in gene expression, cell survival properties, and functional ability compared with those isolated from healthy individuals [80,81]. Similarly, mobilisation of CD34^+^ cells by granulocyte colony stimulating factor (G-CSF) was found to be completely impaired in diabetic patients, whereas the levels of CD34^+^ cells were increased 2.2-fold after mobilisation in non-diabetics [82]. However, some important questions remain unanswered regarding, for example, the effect of the severity of the disease, the time since the acute event, and the duration of pre-existing conditions such as diabetes and hypertension on the number and efficacy of stem cells. In addition to these factors, stem cells from the same organ source might show functional differences depending on the location. For example, CSCs isolated from the atria and the ventricles might have differential gene expression and functional efficacies similar to cardiomyocytes from these two regions [73]. Further, gender differences in the aging process of the human heart might also occur in terms of differences in CSC quality [83]. This evidence suggests the need for more research to develop a personalised evaluation protocol in order to identify optimal stem cell sources depending on patient need.

### Challenges in isolation and expansion of stem cells

The next challenge in translating stem cell therapy from a preclinical to a clinical setting is the practicality of its use, such as the ease and efficiency of its isolation and expansion. Peripheral blood samples are easy to collect, although the relative expansion abilities of the EPCs from the peripheral blood are limited [80]. In contrast, endomyocardial biopsies are difficult to obtain, but CSCs can be isolated and expanded from tiny (approximately 5 mg) endomyocardial biopsies [84]. Within the heart, CSCs from a particular location may be more effective (CSCs from ventricular biopsies) in myocardial regeneration but can be difficult to procure.

Another tricky task in stem cell therapy is marker-based selection. Selection of the right marker for isolation is a critical decision because of the heterogeneity in stem cells such as CSCs [85]. In addition, stem cells from the same source can express heterogeneous markers. Some stem cell markers represent a ‘moving target’, which means that cells retain stem cell-like properties even after losing the expression of these markers following subsequent passages in culture. Hence, capturing stem cells based on these markers becomes illusive at times [86]. As above, the severity of the disease, associated pre-existing conditions, and age also change the expression of stem cell surface markers and the overall number of stem cells expressing a particular marker. For example, the percentage of c-kit^+^ cells was found to be higher in patients with end-stage heart failure compared with patients without end-stage heart failure, whereas the number of circulating endothelial progenitor cells was found to decrease with age [87,88]. The percentage of c-kit was found to be very low in unfractionated CDCs, while almost 90% of these cells were found to be positive for MSC markers [57]. While the suitability of c-kit to identify CSCs has been questioned, the above evidence implies that a single marker may not be sufficient for identifying an effective cell population [74]. This is further supported by a study from Li and colleagues [64], who demonstrated the superior effect of unsorted CDCs compared with a purified c-kit subpopulation. Further studies are required to understand the best marker(s).

In addition to source and marker, isolation technique and culture conditions also offer a major challenge in the expansion of stem cells. Studies suggest that differences in the intensity of enzymatic digestion may affect the type of cells isolated [89]. Different cell types, growth conditions, passaging methods, and number of cell passages considered also influence the outcomes. Pfister and colleagues attributed the low expression of c-kit in CDCs to enzymatic cleavage during the digestion process, as treatment of c-kit^+^ bone marrow cells with a cardiac digestion regimen resulted in a significant reduction in c-kit^+^ cells [90]. One other study showed that CD34^+^ HSCs undergo epigenetic changes involving DNA methylation, which leads to the loss of stemness over subsequent *in vitro* culture passages [91].

The need for increased numbers of appropriate cells continues to limit the clinical development of cell therapy. This has led to a considerable number of studies focussing on *ex vivo* stem cell expansion. Different strategies have been used for the *ex vivo* expansion of stem cells from different sources. A high degree of logistic support may be required for the large scale expansion of stem cells. Open-system configurations such as culture dishes and flasks and closed-systems such as gas-permeable bags, stirred/spinner flasks, flatbed perfusion bioreactors, and three-dimensional scaffolds have been used for culturing and expanding stem cells on a large scale [92]. Amplification of a cell’s proliferation potential is also required. For example, HSCs can be expanded *ex vivo* with the use of specific growth factors and a serum-free media. Some of these factors include thrombopoietin, stem cell factor, G-CSF, IL-6, and Fms-related tyrosine kinase 3 ligand [93]. EPCs are expanded by culturing them on fibronectin in the presence of growth factors favouring endothelial cell growth [94]. Fibroblast growth factors have been used for expanding MSCs and CSCs [65].

The use of growth factors and favourable culture conditions to expand stem cells may also have an effect on their functional properties [95,96]. Reports suggest that the use of basic fibroblast growth factor to culture MSCs can extend the doublings of these cells up to 80 population doublings [96,97]. But as the cells reach senescence, there is a down-regulation of growth factor receptors, and hence they may become resistant to the proliferation stimuli [98,99]. While different combinations of growth factors have been used to improve the proliferation and functional efficacy of stem cells, due to the effect of cell culture conditions on cell physiology it is difficult to compare and interpret the results from these studies [100].

#### Stem cell preconditioning strategies

‘Preconditioning’ refers to any pharmacological, environmental or genetic modification of the cells for the amplification of their potency. Preconditioning promotes stem cell survival and may promote proliferation, differentiation, or its resistance against oxidative stress *in vitro* and *in vivo* [101].

While several studies, including ours, have exclusively confirmed the differentiation and proliferative ability of stem cells in *in vitro* settings, these effects are barely seen after transplantation *in vivo*, with less than 3% of injected cells surviving 1 week after transplantation [102]. To overcome this challenge, combinations of molecular approaches, such as chemical and hypoxic preconditioning and genetic engineering, have been used in an attempt to boost the ability of transplanted cells to withstand the adverse microenvironment, thereby improving their regenerative capacity *in vivo* [47].

Some of the preconditioning techniques include exposing the stem cells to hypoxia, treatment with growth factors and anti-aging compounds, irradiation, and modification of the cells using microRNAs [79,101]. We recently showed that transplanted human pericyte progenitor cells repair the infarcted heart through the activation of an angiogenic programme involving miR-132 [103]. Hence, targeting the expression of microRNAs can be a novel possible approach for enhancing the angiogenic potential of stem cells. Most of these approaches are aimed at salvaging depleted stem cell function. In one of these studies, preconditioning of diabetic MSCs with cardiomyocyte conditioned medium markedly improved their efficacy after transplantation into the diabetic heart [104]. In another study, exposing MSCs to SDF-1 significantly enhanced cell survival, proliferation, and engraftment of the transplanted cells into the infarcted myocardium via SDF-1/CXCR4 signalling [105]. Of note, most of the autologous transplantations are performed in elderly patients with pre-existing diseases; as described above, the functional efficacy of stem cells in these cases are often impaired. Hence, a cell rejuvenation strategy in the form of preconditioning might be required to salvage the therapeutic potential of these cells [80]. In support of this notion, stem cell factor was recently shown to reverse the senescence of cardiac stem cells in the aging myocardium [78].

Data from individual studies aimed at modulating a single target protein or a set of target proteins within a stem cell are inadequate to select the perfect preconditioning strategy. There is thus a need for a high throughput screening plan that can be utilised to identify the whole array of pro-survival and angiogenic target proteins which can be used as targets for stem cell preconditioning.

#### Stem cell delivery to the patient

Finally, when it comes to the delivery of stem cells to a patient, three important factors need to be considered: first is the type and nature of the injury, second the timing of the therapy, and third the ability of the cells to engraft to the host myocardium.

The decision to select a particular cell type should be influenced mainly by the nature of the injury. For example, in cases of chronic ischaemia with a functional myocardium, the purpose is to rescue the non-necrotic ischaemic myocardium and improve the blood supply to it. Hence, it is desirable to use a cell type that secretes pro-angiogenic factors, such as BMCs, or cells that induce vascular regeneration, such as EPCs. On the other hand, if the goal is to regenerate an infarcted myocardium with severe loss of functional myocardial tissue, it would be more suitable to use a progenitor cell type, such as CSCs with cardiomyogenic and vasculogenic capability, or consider the delivery of functional ESC-CMs or iPSC-derived cardiomyocytes [106].

As in other cases, a huge dilemma still remains regarding the best time for stem cell delivery to patients. A comparison of two large clinical trials (LateTIME and Reinfusion of Enriched Progenitor Cells and Infarct Remodelling in Acute Myocardial Infarction (REPAIR-AMI)) in which patients underwent intracoronary infusion of BMCs either 3 to 5 days (REPAIR-AMI) or 2 to 3 weeks (LateTIME) after an acute event showed significant enhancement of LVEF only in patients who received BMCs within 5 days after the acute event [107,108]. This suggests that cell therapy may be efficacious only if administered early after acute MI, which is impossible in the case of autologous transplantation using CSCs. However, the SCIPIO and CADUCEUS trials have demonstrated benefits in patients even when cell therapy was initiated 4 months and 2 to 4 weeks, respectively, after an acute event, although it is not clear if this benefit overweighs the benefit of the REPAIR-AMI trial [66,69]. The researchers who designed the SCIPIO trial argue that cell therapy was initiated after 4 months to separate the effects of CSCs from those of surgical revascularisation immediately after therapy, as in many patients LVEF is known to improve spontaneously during the first few months after CABG surgery [109]. However, due to the lack of immune reactions with CSCs, they can be embraced as an off-the-shelf product if future studies confirm that an early time point could be ideal for stem cell transplantation [110].

In addition to choosing the best stem cell type and the best timing for the treatment, the most important factor after transplantation is the ability of the cells to engraft into the host myocardium. Several studies have reported that cellular engraftment after transplantation into damaged tissues is inadequate and that transplanted cells are susceptible to the hostile ischaemic environment and tend to disappear within a few days [111]. As discussed above, several ongoing research studies have used different methods to precondition stem cells prior to transplantation in order to increase their potential to withstand the adverse microenvironment following transplantation, with the aim of improving their engraftment and survival.

Direct cell delivery into the myocardium has been shown to have disadvantages in terms of meagre cell engraftment and poor mechano-electrical coupling. Cardiac regeneration approaches are evolving from cell therapy to advanced tissue engineering. Cardiac tissue engineering is an alternative strategy in which cell transplantation is accompanied by the support of biomaterials (called scaffolds) and regulatory factors (for example, growth factors) [112]. The *in vitro* engineering of beating cardiomyocyte-containing tissue constructs and the engineering of stem cell-containing tissue constructs are the two strategies currently used for cell implantation. The cardiac tissue constructs are formed by seeding cardiomyocytes in three-dimensional scaffolds and culturing under appropriate conditions to develop cell alignment, electrical communication, and spontaneous beating *in vitro* [113]. Apart from using the tissue constructs of beating cardiomyocytes, as indicated above, engineered stem cell sheets are also an attractive choice for therapeutic delivery, owing to their paracrine effects and plasticity [114]. In the future, these bio-engineered cardiac patches or cell sheets may become the preferred approach for delivering stem cells into the diseased heart [51,115].

## Conclusion

While stem cell therapy has the potential to become a next-generation treatment, several hurdles need to be overcome before it becomes a routine therapy for cardiovascular regeneration. In order to achieve this, it is necessary to design studies that can systematically compare different cell types at the same time point with minimal variability in type of disease, age, gender, and pre-existing conditions.

## Abbreviations

BMC: Bone marrow-derived cell
CABG: Coronary artery bypass graft
CADUCEUS: CArdiosphere-Derived aUtologous stem CElls to reverse ventricUlar dySfunction
CDC: Cardiosphere-derived cell
CSC: Cardiac stem cell
CXCR4: C-X-C chemokine receptor type 4
EPC: Endothelial progenitor cell
ESC: Embryonic stem cell
ESC-CM: ESC-derived cardiomyocyte
G-CSF: Granulocyte colony stimulating factor
HSC: Hematopoietic stem cell
IHD: Ischaemic heart disease
IL: Interleukin
iPSC: Induced pluripotent stem cell
LVEF: Left ventricular ejection fraction
MI: Myocardial infarction
MSC: Mesenchymal stem cell
REPAIR-AMI: Reinfusion of enriched progenitor cells and infarct remodelling in acute myocardial infarction
SCIPIO: Stem cell Infusion in patients with Ischemic Cardiomyopathy
SDF-1: Stromal cell-derived factor-1
